# The Soluble Form of CTLA-4 from Serum of Patients with Autoimmune Diseases Regulates T-Cell Responses

**DOI:** 10.1155/2014/215763

**Published:** 2014-01-29

**Authors:** Rita Simone, Giampaola Pesce, Princey Antola, Margarita Rumbullaku, Marcello Bagnasco, Nicola Bizzaro, Daniele Saverino

**Affiliations:** ^1^Section of Human Anatomy, Department of Experimental Medicine, University of Genoa, Via De Toni 14, 16132 Genoa, Italy; ^2^Departments of Medicine and Cell Biology, North Shore University Hospital, Manhasset, NY 11030, USA; ^3^Autoimmunity Unit, Department of Internal Medicine, University of Genoa, 16132 Genoa, Italy; ^4^Laboratory of Clinical Pathology, San Antonio Hospital, Tolmezzo, 33100 Udine, Italy

## Abstract

Cytotoxic T lymphocyte associated antigen-4 (CTLA-4) is a costimulatory receptor transducing a potent inhibitory signal. Increasing evidence showed that CTLA-4 gene is an important susceptibility locus for autoimmune disorders. Alternatively spliced mRNA generates a soluble form, called sCTLA-4. Whereas low levels of sCTLA-4 are detected in normal human serum, increased/high serum levels are observed in several autoimmune diseases. The biological significance of increased sCTLA-4 serum level is not fully clarified yet. It can be envisaged that sCTLA-4 specifically inhibits the early T-cell activation by blocking the interaction of CD80/CD86 with the costimulatory receptor CD28. On the other hand, higher levels of sCTLA-4 could contend the binding of the membrane form of CTLA-4 with CD80/CD86, in later activation phase, causing a reduction of inhibitory signalling. We showed that sCTLA-4 from sera of patients with different autoimmune diseases is able to display functional activities on an *in vitro* system acting on the proliferation capability and modulating the secretion of cytokines. We observed a dual effect of sCTLA-4: inhibiting the secretion of IFN-**γ**, IL-2, IL-7, and IL-13 and activating the secretion of TGF-**β** and IL-10. This study underlines the role of sCTLA-4 in modulating the immune response and its relevance in autoimmune disease pathogenesis.

## 1. Introduction

T-cell activation is a result of two phases: the first signal is delivered by the antigenic peptide presented by major histocompatibility complex molecules, and the second one (costimulatory signal) is mediated by CD28 interaction with B7 family members on antigen presenting cells [1]. Cytotoxic T lymphocyte associated gene-4 (CTLA-4) is a type I glycoprotein on the surface of activated T-cells [2]. CTLA-4 is a member of the Ig gene superfamily and along with its homologue, CD28, is a B7 binding protein [3, 4]. The function of CTLA-4 is to attenuate the ongoing immune response [5, 6]. The most convincing data that supports such a role for CTLA-4 comes from experiments in which the CTLA-4 gene is inactivated by a construction of CTLA-4 knockout mice [7, 8]. These mice demonstrate profound polyclonal lymphoproliferative disorders that infiltrate most major organ systems and die a few weeks after birth. The majority of animals has increased levels of IgG; this fact illustrates the role of CTLA-4 on humoral immune responses as well [9, 10]. A role for CTLA-4 in autoimmune diseases is suggested by the observation that blockade of B7/CTLA-4 interaction via administration of anti-CTLA-4 mAb exacerbates autoimmune diseases in animal models such as experimental autoimmune encephalomyelitis [11] and type 1 diabetes (T1D) [12, 13].

It has been demonstrated that CTLA-4 is able to generate messenger RNA (mRNA) for two known isoforms: a full-length isoform (flCTLA-4) encoded by exon 1 (leader peptide), exon 2 (ligand binding domain), exon 3 (transmembrane domain), and exon 4 (cytoplasmic tail) and a soluble form (sCTLA-4), which lacks exon 3. sCTLA-4, originating from alternative splicing, results in the loss of a cysteine residue and is found in the serum as a soluble monomeric protein [14–16]. The presence of high concentrations of sCTLA-4 was observed in sera of patients with autoimmune thyroid diseases [16, 17], as well as in patients with other autoimmune diseases, such as type 1 diabetes, diffuse cutaneous systemic sclerosis [18], systemic lupus erythematosus [19], myasthenia gravis [20], and celiac disease ([21], reviewed in [22]). In addition, plasma levels of sCTLA-4 are increased in patients with allergic asthma [23] and allergy to hymenoptera venom [24] and in B-ALL [25].

The present study has been conceived to further evaluate the immunopathological roles of sCTLA-4. In particular, we have investigated the functional activities of sCTLA-4 observing its inhibitory role in T-cell proliferation induced by bidirectional mixed lymphocyte reaction and its ability to modulate the secretion of cytokines (such as IFN-*γ*, IL-2, IL-13, IL-7, TGF-*β*, and IL-10).

## 2. Materials and Methods

### 2.1. Serum Samples

**Figure 1:**
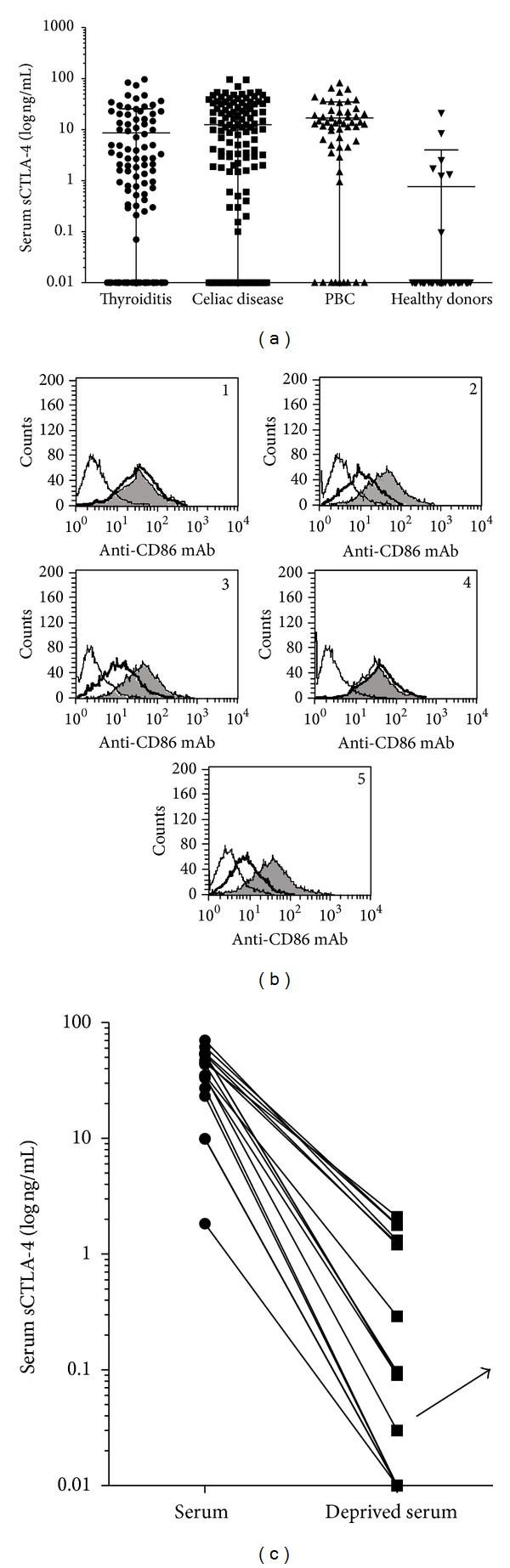
A soluble form of CTLA-4 is differently found in serum of patients with autoimmune diseases and normal donors. (a) A soluble form of CTLA-4 is found in serum of patients with different autoimmune diseases. The concentration of sCTLA-4 was evaluated by ELISA on sera collected from AIT (*n* = 90), CD (*n* = 160), and PBC patients (*n* = 70) and a healthy donor group as a control (*n* = 50). Results are expressed as log of ng/mL. Each sample was diluted 1 : 10 and tested in triplicate. Deviation between triplicates was <10% for any reported value (as previously published [17, 21]). Lines indicate the mean values for each group. The differences among the different groups of patients and the healthy donors were determined by non-parametric Mann-Whitney Rank Sum test (*P* < 0.001). (b) Data from three independent experiments showing the capability of sCTLA-4 to inhibit anti-CD86 mAb immunoreactivity. The test was performed utilizing a THP-1 cell line expressing CD86 molecules. Three different representative sera from patients with autoimmune disease (21AIT negative, plot 1; 55AIT, plot 2, and 101CD, plot 3, positive for sCTLA-4; and 101CD-deprived sera, plot 4) were preincubated with THP-1 cells prior to stain cells with mAb to CD86. The addition of CTLA-4 Ig fusion protein was utilized as positive control of interference of specific mAb binding to CD86, plot 5. Thinner lines represent controls with a not reacting mAb, thicker lines represent the inhibitory effect of serum sCTLA-4 (and CTLA-4 Ig, plot 5) on anti-CD86 mAb immunoreaction, and shaded curves represent the immunostaining of untreated cells with anti-CD86 mAb. (c) The sera of 15 autoimmune patients with high concentrations of sCTLA-4 were depleted to show that sCTLA-4 is the protein of serum with functional activities (see Figures 3 and 4). Results are expressed as log of ng/mL. Each sample was diluted 1 : 10 and tested in triplicate. The arrow indicates the result of *in vitro* depletion of sCTLA-4 on 101CD serum (used also for the experiment shown in (b), plot 4).

In previous studies we have evaluated 320 patients with autoimmune diseases (90 autoimmune thyroid disease, AIT, 160 celiac disease, CD, and 70 primary biliary cirrhosis, PBC), who showed elevated sCTLA-4 levels as measured by ELISA (see the following) (see Figure 1) ([17], [21], and unpublished results). For the present study, we have selected 15 sera (5AIT, 5CD and 5PBC), which displayed high concentrations of sCTLA-4, and 6 sera (2AIT, 2CD, and 2PBC) resulted negative for sCTLA-4. For control, we collected sera from 45 age- and sex-matched healthy donors, as previously described [17, 21].

### 2.2. ELISA

Specific ELISA kits were used for measuring serum sCTLA-4 levels (Bender Medsystems, Milano, Italy), according to the manufacturer's protocol. Each sample was diluted 1 : 10 and tested in triplicate. Deviation between triplicates was <10% for any reported value. The lowest sensitivity threshold is 0.1 ng/mL.

The analytical response was linear approximately between 0.162 and 1.200 of absorbance values (corresponding to 0.1–50 ng/mL) as assessed by serial dilution test using a strongly positive serum (data not shown) [17, 21].

### 2.3. Mixed Leukocyte Reactions (MLR)

The cells used in these experiments were from a panel of previously HLA typed laboratory volunteers and were selected to provide two HLA-DR mismatches. PBMCs were isolated by standard Ficoll-Hypaque density gradient centrifugation, and equal numbers of cells (200,000) were cocultured in tissue culture medium (RPMI 1640 supplemented with 10% human AB serum, 50 IU/mL penicillin-streptomycin, and 50 mM 2-mercaptoethanol) containing graded dilutions of sCTLA-4 test sample. Sera were added at the beginning of the experiments. Triplicate cultures were set up in 0.2 mL volumes in flat-bottom 96-well microculture plates and were incubated for 5 days at 37°C and 5% CO_2_ in air. The cultures were pulsed with 0.5 mCi ^3^H-thymidine and harvested 18 h later. Isotope incorporation was measured on a Beckman *β*-counter. CTLA-4 Ig (provided by A. Lanzavecchia, Institute for Research in Biomedicine, Bellinzona, Switzerland) was utilized as a positive control of molecule reacting with CD80 and CD86 ligands and transducing an inhibitory signal.

### 2.4. Depletion of sCTLA-4 Protein from Sera

In order to understand the effective role played by serum sCTLA-4 avoiding the effect related to other serum proteins, we used sera previously depleted of sCTLA-4 in MLR. To this end we incubated the sera from patients positive for sCTLA-4 after ELISA testing for 3 hrs at room temperature on ELISA plates coated with the capture mAb anti-sCTLA-4 (included in the ELISA kit). This procedure was repeated twice before utilizing these sera in a MLR assay as specified before. In addition, after the depletion procedure sera were tested in ELISA in order to verify the absence of sCTLA-4. As a control we used sera not subjected to the depletion procedure.

### 2.5. Cytokines Measurement

The levels of interferon-*γ* (IFN-*γ*), interleukin-2 (IL-2), IL-13, IL-7, IL-10, and transforming growth factor-*β* (TGF-*β*) released in the supernatant during the MLR tests were determined using commercially available ELISA kits (Bender Medsystems).

### 2.6. Statistical Analysis

Statistical analysis was performed using the Mann-Whitney *U* test for comparison of sCTLA-4 levels by using the GraphPad Prism4 software 4.0 (GraphPad Software Inc., CA, USA). Differences in cell proliferation between control and mAb-treated cells, as well as cytokines secretion, were observed. To assess their statistical significance, the Student's *t*-test was used with a level of *P* < 0.05.

## 3. Results

### 3.1. Serum sCTLA-4 Levels in Autoimmune Patients and Normal Donors

To measure the presence of a circulating form of CTLA-4 in human serum, we utilized a sensitive ELISA, as previously published [17, 21].  Figure 1(a) shows combined data from the ELISA on patients with AIT and CD and normal healthy volunteers, summarising our previous studies where the presence of sCTLA-4 was evaluated in 250 patients with autoimmune diseases (90AIT and 160CD [17, 21]. Preliminary unpublished results on 70PBC are also included. sCTLA-4 was virtually undetectable in the very large majority of healthy volunteers as defined by the detection limit of 0.1 ng/mL for this assay. Six serum samples out of 50 from healthy controls had detectable sCTLA-4 (from 0.1 to 21.06 ng/mL). By contrast, 70 out of 90AIT patients (78%, from 0.1 to 96.50 ng/mL), 100 out 160CD patients (62.5%, from 0.1 to 96.04 ng/mL) and 60 out 70PBC patients (82.9%, from 0.1 to 81.79) had detectable circulating CTLA-4 levels (*P* < 0.05) (Figure 1(a)). Of note, no significative differences among autoimmune patients were observed. Finally, there was no obvious relationship between the sex and either age of the patient or severity of autoimmune disease and levels of sCTLA-4.

In order to verify the capability of sCTLA-4 to bind its natural ligands expressed on antigen presenting cells and to avoid the possibility that the soluble molecule is the result of a degradation or shedding process, we performed a flow cytometry-based binding assay (Table 1). Pretreatment of THP-1 cell lines with sera from autoimmune patients characterized by the presence of sCTLA-4 led to a significant reduction of staining by anti-CD86 mAb (12AIT, 26CD, and 31PBC). These results were obtained on two independent serum samples and ranged from 56% to 85% of inhibition, for CD86 (Figure 1(b)). A negligible inhibition (<5%) was observed when sera negative for sCTLA-4 were tested (Figure 1(b)). CTLA-4 Ig, which reduced the staining of THP-1 cell lines by anti-CD86 mAb, was utilized as a positive control (Figure 1(b)). These data show that the CTLA-4 immunoreactive material present in human serum represents an intact functional receptor for CD86 ligand (Figure 1(b)).

Finally, in order to show that sCTLA-4 is the only serum protein able to bind B7 molecules, we have performed a similar experiment using sera previously deprived of sCTLA-4 proteins as described in Section 2. Controls were the same sera before depletion. As shown in Figure 1(b), the serum deprived of sCTLA-4 did not significantly reduce the staining of THP-1 cells. The confirmation of the deprivation procedure is shown in Figure 1(c).

### 3.2. sCTLA-4 from Serum Autoimmune Patients Is Able to Inhibit T-cell Proliferation Induced by Bidirectional Mixed Lymphocyte Reaction (MLR)

**Figure 2:**
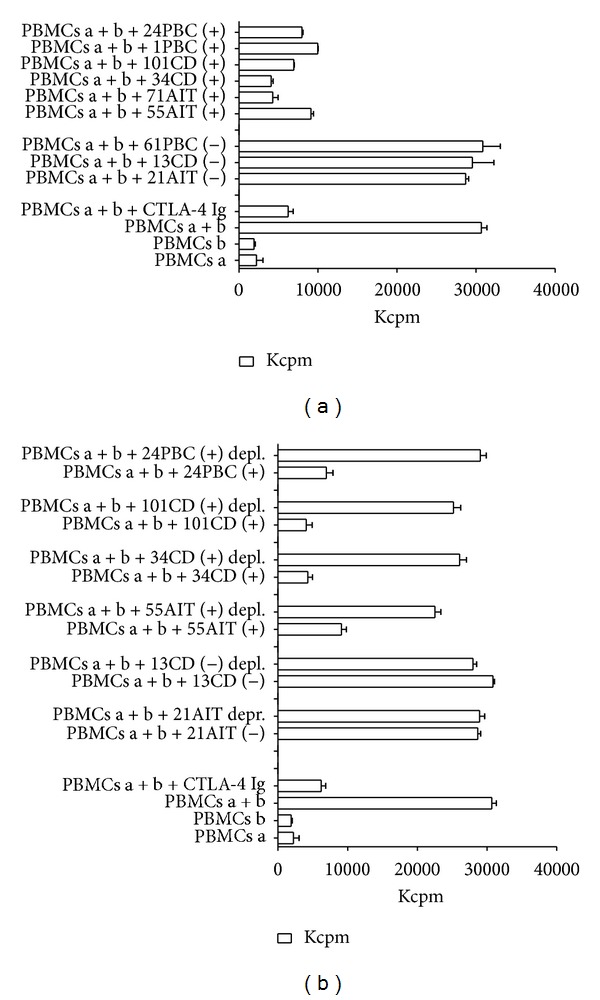
Immunoregulatory properties of sCTLA-4. (a) sCTLA-4 containing sera from autoimmune patients (55AIT, 71AIT, 34CD, 101CD, 1PBC, and 24PBC) are able to inhibit significatively a MLR. CTLA-4 Ig fusion protein is a positive control of inhibition of proliferation. Sera from autoimmune patients negative for sCTLA-4 (21AIT, 13CD, and 61PBC) do not affect the proliferation rate. The data represent the mean of quadruplicate samples. The averages ± standard deviations (error bars) of three different experiments are shown. Positive or negative sera are indicated by an alphanumeric code followed by (+) and (−), respectively. (b) We show that the inhibitory effect on T lymphocytes proliferation is related to sCTLA-4 and is not due to a different serum protein, by using sCTLA-4-deprived sera (55AIT, 34CD, 101CD, and 24PBC). As depicted from the graphic, depleted sera are unable to alter significatively the proliferation in this assay. Controls are the same sera before the treatment with the capture mAb specific for sCTLA-4 (see Section 2). In addition, sera resulted to be negative for sCTLA-4 (21AIT and 13CD) following the deprivation method did not change their inability to affect proliferation. A representative experiment out of five is shown.

We examined the immunoregulatory properties of sCTLA-4 on MLR. The data presented in Figure 2(a) show that sCTLA-4 had significant inhibitory effect, similarly to that obtained by the addition of CTLA-4 Ig. We verified the dose effect of sCTLA-4 in sera of autoimmune patients. We choose four different positive sera (namely, 55AIT, 71AIT, 34CD, 101CD, 1PBC, and 24PBC, with the concentration of sCTLA-4, respectively, 36.4, 73.8, 96.04, 53.74, 33.63, and 43.33 ng/mL) and 3 sera (namely, 21AIT, 13CD, and 61PBC) negative for sCTLA-4 (<0.1 ng/mL, lower then the threshold limits of the ELISA). As shown in Figure 2(a), the inhibition rate depends on serum concentration of sCTLA-4. In the same way the sera from controls, containing undetectable sCTLA-4, were tested to analyse the rate of inhibitions. As shown in Figure 2(a), sera negative for sCTLA-4 were ineffective on the proliferative rate. Thus, we can suggest the functional role of serum sCTLA-4 in modulating T-cell activity.

Finally, in order to underline the functional effect of sCTLA-4, the sera of autoimmune patients with high concentration of sCTLA-4 tested by ELISA were sCTLA4-deprived (Figure 1(c)) and then used in the MLR test (Figure 2(b)). Controls were the same sera before depletion. The results show that the deprived sera are ineffective on MLR ^3^H-thymidine incorporation. This strongly suggests that sCTLA-4 is a major serum protein able to inhibit the proliferation and the intervention of putative different serum molecules can be reasonably ruled out.

### 3.3. sCTLA-4 from Serum of Autoimmune Patients Is Able to Decrease the Levels of IFN-*γ*, IL-2, and IL-13

**Figure 3:**
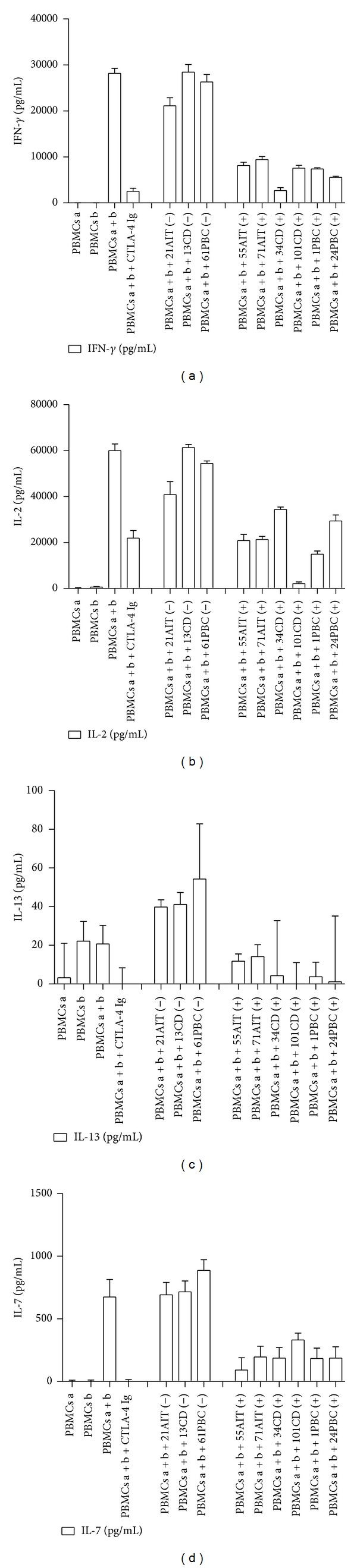
sCTLA-4 from autoimmune sera is able to decrease the secretion of activator cytokines. Soluble CTLA-4 containing sera from autoimmune patients are able to decrease the production of interferon-*γ* (IFN-*γ*), interleukin-2 (IL-2), IL-13, and IL-7. These data are confirmed by control experiment with sera of autoimmune patients that were negative for sCTLA-4 through ELISA test. Positive or negative sera are indicated by an alphanumeric code followed by (+) and (−), respectively. CTLA-4 Ig fusion protein is a positive control of secretion of cytokines. The concentration of activator cytokines is expressed in pg/mL. The data represent the averages ± standard deviations (error bars) of double samples. A representative experiment out of three is shown.

We can hypothesise that the addition of sCTLA-4 to MLR test not only regulates the proliferative response, but also modulate cytokine production. For this reason the supernatant of cultured PBMCs during the MLR test was analysed using available ELISA kits. We found that the presence in the culture media of sera with high levels of sCTLA-4 (namely, 55AIT, 71AIT, 34CD, 101CD, 1PBC, and 24PBC) determines a decrease of production of IFN-*γ*, IL-2, IL-13, and IL-7. In detail, the addition of these sera yielded a decrease of cytokine production varying from 71.2 to 90.4% for IFN-*γ*, from 42.7 to 96.5% for IL-2, from 31.9 to 100% for IL-13, and from 50.8 to 86.3% for IL-7. These effects were similar to those obtained by the action of CTLA-4 Ig. Otherwise, sera negative for sCTLA-4 (namely, 21AIT, 13CD, and 61PBC) did not modify the production of these activator cytokines (Figure 3).

### 3.4. sCTLA-4 from Serum of Autoimmune Patients Is Able to Increase the Levels of TGF-*β* and IL-10

**Figure 4:**
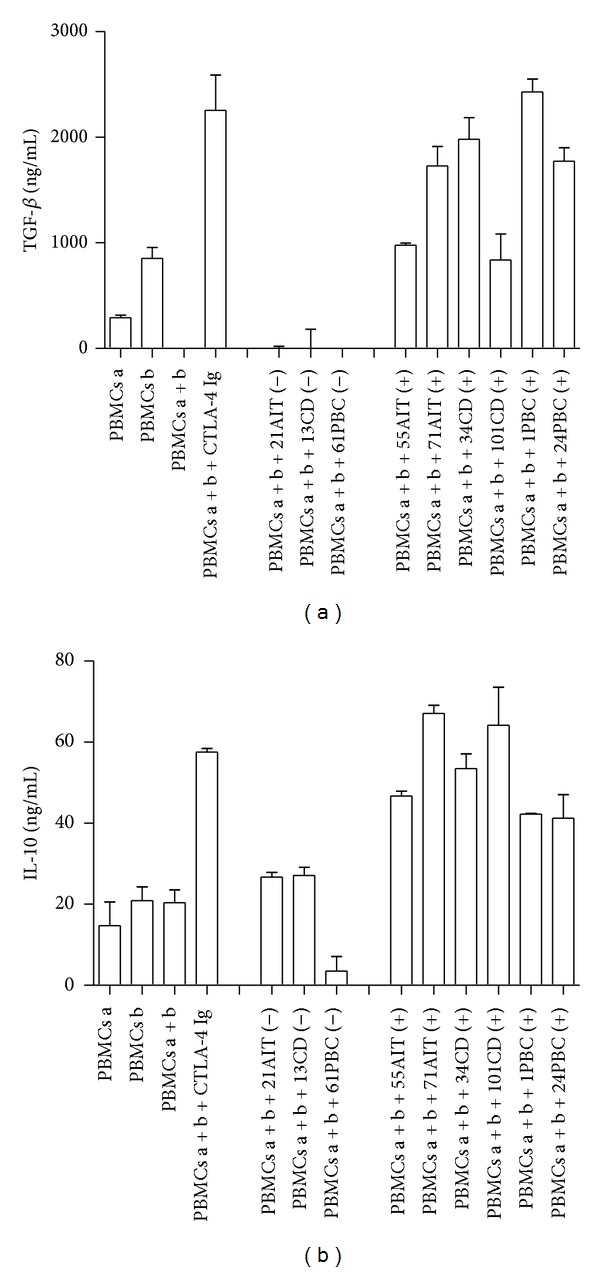
sCTLA-4 from autoimmune sera is able to increase the secretion of inhibitory cytokines. We show the ability of sCTLA-4 contained in the sera of autoimmune patients to increase the secretion of transforming growth factor-*β* (TGF-*β*) and Interleukin-10 (IL-10). Positive or negative sera are indicated by an alphanumeric code followed by (+) and (−), respectively. CTLA-4 Ig fusion protein is a positive control of secretion of cytokines. The concentration of inhibitory cytokines is expressed in ng/mL. The data represent the averages ± standard deviations (error bars) of double samples. A representative experiment out three is shown.

In the same experimental condition, we evaluated the production of TGF-*β* and IL-10 for their well-known inhibitory properties on T-cell proliferation. As shown in Figure 4, we found that sera positive for sCTLA-4 (namely, 55AIT, 71AIT, 34CD, 101CD, 1PBC, and 24PBC) are able to increase the levels of TGF-*β* and IL-10, while the negative ones (namely, 21AIT, 13CD, and 61PBC) are ineffective on the secretion of these cytokines. Thus, we can conclude that sCTLA-4 possesses a dual effect on cytokine production, finely modulating the balance of activator and inhibitory cytokines during the ongoing of the immune response.

## 4. Discussion

The regulation of immune responses is the outcome of a balance between positive signals that trigger them and inhibitory mechanisms that prevent excessive clonal expansion and autoimmunity [1–6, 26, 27]. Therefore, a prevalence of activation should render T-cells responsive to antigens, whereas a prevalence of inhibition should lead to T-cell anergy. The majority of the data on the inhibitory function exerted by CTLA-4 has been gathered by studies of proliferation or cytokine production of T lymphocytes analysing the interaction between CTLA-4 and CD80/CD86 at the membrane level [1–6, 26, 27]. Most recently, the central role of CTLA-4 in modulating the immune response was reanalysed hypothesising the “reverse stop-signal model” [28]. By reversing the TCR induced stop signal for T-cell motility, this model proposes that CTLA-4 limits the dwell time between T-cells and antigen presenting cells (APCs), thereby reducing the level of T-cell activation. The reverse stop-signal model can potentially explain how CTLA-4 regulates the activation threshold of T-cells, anergy, autoimmunity, tissue infiltration, and various T-cell effector functions [28]. Because of its inhibitory role, CTLA-4 is a strong candidate susceptibility gene in autoimmunity and several studies suggest disease associated polymorphisms [22]. Years ago Oaks and Hallett [16] have described for the first time an alternate transcript of the CTLA-4 gene that encodes a protein that lacks a transmembrane region and likely represents a native soluble form of CTLA-4 (sCTLA-4). Subsequently, the expression of sCTLA-4 was found in several autoimmune pathologies as autoimmune thyroid disease [16, 17], in systemic sclerosis [18], systemic lupus erythematosus [19], myasthenia gravis [20], and celiac disease ([21], revised in [22]). In addition, sCTLA-4 has been found in sera from allergic asthma [23] and allergy to hymenoptera venom [24] and in pediatric B-ALL patients [25].

In this study we used previously published data demonstrating the presence of sCTLA-4 immunoreactive material in the sera from different patients with autoimmune diseases [17, 21] and extend these findings showing a possible functional role of this soluble molecule. Of interest, we found an increase of sera sCTLA-4 in autoimmune diseases when compared not only to healthy donors, but also to nonautoimmune diseases [17, 21]. Thus, we can postulate a pathogenetic role of this soluble molecule preferentially related to autoimmune disease. In addition, the capability of sCTLA-4 to react with its known ligands (namely, CD80 and CD86), similarly to the synthetic receptor (CTLA-4 Ig), supports the notion that sCTLA-4 is a functional receptor. Furthermore, its ability to interfere with T-cell proliferation in MLR supports its possible immunoregulatory action *in vivo*.

To underline the role of sCTLA-4 in modulating the immune response, we verified its ability to modulate cytokine production. Cytokines are soluble factors able to recruit CD4^+^ T lymphocytes and B cells so as to begin the immune response. In particular, we verified the effect of sCTLA-4 on the secretion of IFN-*γ* [29, 30] that sustains the response of T-cells to mitogens and Ag and that synergises with IL-2 and promotes the expression of IL-2R on the membrane of T lymphocytes. IL-2 [31] is a well-known non-Ag-specific proliferation factor for T-cells that prompts cell cycle progression of resting cells, thus allowing clonal expansion of activated T lymphocytes. IL-13 [32, 33] acts in concert with IL-2 for the regulation of IFN-*γ* synthesis. IL-7 is an essential survival factor for lymphocytes. However, recent studies revealed a much more sophisticated role of this cytokine in fine-tuning T-cell functions and its dysregulation seems to be involved in pathogenesis of autoimmune diseases [34]. IL-10 [35] inhibits Ag- or anti-CD3-induced proliferation of T-cells and downregulates the production of IL-2 and IFN-*γ*. Finally, TGF-*β* [36, 37] inhibits the proliferation of T lymphocytes mainly by reducing IL-2-mediated signals. In all experiments, a modulatory role of sCTLA-4 has been demonstrated. Of note, the production of IL-2, IFN-*γ*, IL-13, and IL-7 was sharply reduced following addition of serum sCTLA-4; in contrast, the production of IL-10 and TGF-*β* was significantly increased. It follows that the outcome of sCTLA-4, at least *in vitro*, results in a control of cell-mediated immunity. Our observations suggest that the soluble form of CTLA-4 plays a dual role. In our experimental model, the induction of IL-10 and TGF-*β* production could suggest an additional role of sCTLA-4 in the regulation of the intensity of immune responses. However, it should be noted that the sCTLA-4 effect, at least in our *in vitro* model, is not univocal. In fact, some parameters analysed seem to be more easily inhibited than others. Probably, one has to take into account the biological variability in the different samples analysed or the possible presence of possible different molecules with inhibitory effect.

The clinical picture of autoimmune thyroiditis, as well as celiac disease and primitive biliary cirrhosis, shows anatomohistological changes, with subsets of peripheral blood lymphocytes infiltration [38]. De Carli et al. [39] explained the important role of Th1/Th2 lymphocytes balance in autoimmunity, underlining the role of the activator and inhibitory cytokines secreted by these subsets of lymphocytes. The functional subsets of T lymphocytes are based on profiles of cytokine production. Th1 cells produce IL-2, TNF-*α*, and IFN-*γ* which mediate cellular immunity, macrophage activation, and cytotoxicity and help in B-cell production of opsonizing and complement fixing antibodies. In contrast, Th2 cells, which produce IL-4, IL-5, IL-6, and IL-10, seem to promote humoral and allergic responses [39]. This analysis shows the role of cytokines in modulating the immune response and their role in autoimmunity diseases. Like other soluble receptors [30–40], sCTLA-4 may display immunoregulatory functions. We show that sCTLA-4 is able to decrease the proliferation of T-cells and the production of activator cytokines as IL-2, IL-13 and IFN-*γ* *in vitro* and this regulation may have a clinical feature given that the patients with autoimmune diseases show prominent lymphocyte infiltration and a greater amount of IFN-*γ* [40]. Moreover, the inhibitory signal transduced by sCTLA-4 is able to increase TGF-*β* and IL-10 showing an additional effect in regulating the pathway.

Thus, sCTLA-4 may have important immunoregulatory functions and its effect might depend on the activation state of the cells involved. On resting cells, sCTLA-4 could block CD80/CD86-CD28 interactions, interfering with T-cell costimulation. On the contrary, inhibition of CD80/CD86-CTLA-4 interactions on activated T-cells (when the transmembrane form of CTLA-4 is expressed) may prevent downregulation of T-cell responses. Such a hypothesis is supported by data previously obtained from *in vitro* models [17].

## Figures and Tables

**Table 1:** sCTLA-4 from sera of autoimmune patients is able to interact with its natural ligands.

Patient's serum	% of inhibition	sCTLA-4 (ng/mL)
55AIT	71	36.40
71AIT	82	73.80
12AIT	58	14.71
37AIT	56	23.04
66AIT	64	47.11
26CD	59	32.18
34CD	84	96.04
101CD	79	53.74
77CD	72	52.76
123CD	77	49.59
1PBC	67	33.63
24PBC	79	43.33
31PBC	62	37.93
41PBC	81	61.44
43PBC	85	81.79
21AIT	0.1	<0.1
44AIT	0.5	<0.1
13CD	0.2	<0.1
97CD	0.3	<0.1
61PBC	0.4	<0.1
69PBC	0.5	<0.1
CTLA-4 Ig	99.9	

## References

[1] Lenschow DJ, Walunas TL, Bluestone JA (1996). CD28/B7 system of T cell costimulation. *Annual Review of Immunology*.

[2] Brunet J-F, Denizot F, Luciani M-F (1987). A new member of the immunoglobulin superfamily—CTLA-4. *Nature*.

[3] Linsley PS, Brady W, Grosmaire L, Aruffo A, Damle NK, Ledbetter JA (1991). Binding of the B cell activation antigen B7 to CD28 costimulates T cell proliferation and interleukin 2 mRNA accumulation. *Journal of Experimental Medicine*.

[4] Linsley PS, Brady W, Urnes M, Grosmaire LS, Damle NK, Ledbetter JA (1991). CTLA-4 is a second receptor for the B cell activation antigen B7. *Journal of Experimental Medicine*.

[5] Thompson CB, Allison JP (1997). The emerging role of CTLA-4 as an immune attenuator. *Immunity*.

[6] Saverino D, Tenca C, Zarcone D (1999). CTLA-4 (CD152) inhibits the specific lysis mediated by human cytolytic T lymphocytes in a clonally distributed fashion. *Journal of Immunology*.

[7] Huurman VAL, Unger WWJ, Koeleman BPC (2007). Differential inhibition of autoreactive memory- and alloreactive naive T cell responses by soluble cytotoxic T lymphocyte antigen 4 (sCTLA4), CTLA4Ig and LEA29Y. *Clinical and Experimental Immunology*.

[8] Waterhouse P, Penninger JM, Timms E (1995). Lymphoproliferative disorders with early lethality in mice deficient in Ctla-4. *Science*.

[9] Tivol EA, Borriello F, Schweitzer AN, Lynch WP, Bluestone JA, Sharpe AH (1995). Loss of CTLA-4 leads to massive lymphoproliferation and fatal multiorgan tissue destruction, revealing a critical negative regulatory role of CTLA-4. *Immunity*.

[10] Merlo A, Tenca C, Fais F (2005). Inhibitory receptors CD85j, LAIR-1, and CD152 down-regulate immunoglobulin and cytokine production by human B lymphocytes. *Clinical and Diagnostic Laboratory Immunology*.

[11] Karandikar NJ, Vanderlugt CL, Walunas TL, Miller SD, Bluestone JA (1996). CTLA-4: a negative regulator of autoimmune disease. *Journal of Experimental Medicine*.

[12] Lühder F, Höglund P, Allison JP, Benoist C, Mathis D (1998). Cytotoxic T lymphocyte-associated antigen 4 (CTLA-4) regulates the unfolding of autoimmune diabetes. *Journal of Experimental Medicine*.

[13] Marron MP, Raffel LJ, Garchon H-J (1997). Insulin-dependent diabetes mellitus (IDDM) is associated with CTLA4 polymorphisms in multiple ethnic groups. *Human Molecular Genetics*.

[14] Salomon B, Bluestone JA (2001). Complexities of CD28/B7: CTLA-4 costimulatory pathways in autoimmunity and transplantation. *Annual Review of Immunology*.

[15] Magistrelli G, Jeannin P, Herbault N (1999). A soluble form of CTLA-4 generated by alternative splicing is expressed by nonstimulated human T cells. *European Journal of Immunology*.

[16] Oaks MK, Hallett KM (2000). Cutting edge: a soluble form of CTLA-4 in patients with autoimmune thyroid disease. *Journal of Immunology*.

[17] Saverino D, Brizzolara R, Simone R (2007). Soluble CTLA-4 in autoimmune thyroid diseases: relationship with clinical status and possible role in the immune response dysregulation. *Clinical Immunology*.

[18] Sato S, Fujimoto M, Hasegawa M (2004). Serum soluble CTLA-4 levels are increased in diffuse cutaneous systemic sclerosis. *Rheumatology*.

[19] Wong CK, Lit LCW, Tam LS, Li EK, Lam CWK (2005). Aberrant production of soluble costimulatory molecules CTLA-4, CD28, CD80 and CD86 in patients with systemic lupus erythematosus. *Rheumatology*.

[20] Wang X-B, Kakoulidou M, Giscombe R (2002). Abnormal expression of CTLA-4 by T cells from patients with myasthenia gravis: effect of an AT-rich gene sequence. *Journal of Neuroimmunology*.

[21] Simone R, Brizzolara R, Chiappori A (2009). A functional soluble form of CTLA-4 is present in the serum of celiac patients and correlates with mucosal injury. *International Immunology*.

[22] Schiavo M, Saverino D (2013). The role of CTLA-4 gene polymorphisms in autoimmune disease pathogenesis: a 2012 update. *Immunology, Endocrine & Metabolic Agents in Medicinal Chemistry*.

[23] Wong CK, Lun SWM, Ko FWS, Ip WK, Hui DSC, Lam CWK (2005). Increased expression of plasma and cell surface co-stimulatory molecules CTLA-4, CD28 and CD86 in adult patients with allergic asthma. *Clinical and Experimental Immunology*.

[24] Riccio AM, Saverino D, Pesce G (2012). Effects of different up-dosing regimens for hymenoptera venom immunotherapy on serum CTLA-4 and IL-10. *PLoS ONE*.

[25] Simone R, Tenca C, Fais F (2012). A soluble form of CTLA-4 is present in paediatric patients with acute lymphoblastic leukaemia and correlates with CD1d+ expression. *PLoS ONE*.

[26] Walunas TL, Lenschow DJ, Bakker CY (1994). CTLA-4 can function as a negative regulator of T cell activation. *Immunity*.

[27] Krummel MF, Allison JP (1995). CD28 and CTLA-4 have opposing effects on the response of T cells to stimulation. *Journal of Experimental Medicine*.

[28] Rudd CE (2008). The reverse stop-signal model for CTLA4 function. *Nature Reviews Immunology*.

[29] Gajewski TF, Fitch FW (1988). Anti-proliferative effect of IFN-*γ* in immune regulation. I. IFN-*γ* inhibits the proliferation of Th2 but not Th1 murine helper T lymphocyte clones. *Journal of Immunology*.

[30] Naylor SL, Sakaguchi AY, Shows TB (1983). Human immune interferon gene is located on chromosome 12. *Journal of Experimental Medicine*.

[31] Josimovic-Alasevic O, Herrmann T, Diamantstein T (1988). Demonstration of two distinct forms of released low-affinity type interleukin 2 receptors. *European Journal of Immunology*.

[32] McKenzie ANJ, Culpepper JA, de Waal Malefyt R (1993). Interleukin 13, a T-cell-derived cytokine that regulates human monocyte and B-cell function. *Proceedings of the National Academy of Sciences of the United States of America*.

[33] Minty A, Chalon P, Derocq J-M (1993). Interleukin-13 is a new human lymphokine regulating inflammatory and immune responses. *Nature*.

[34] Dooms H (2013). Interleukin-7: fuel for the autoimmune attack. *Journal of Autoimmunity*.

[35] Nagalakshmi ML, Murphy E, McClanahan T, de Waal Malefyt R (2004). Expression patterns of IL-10 ligand and receptor gene families provide leads for biological characterization. *International Immunopharmacology*.

[36] Lawrence DA (1996). Transforming growth factor-*β*: a general review. *European Cytokine Network*.

[37] del Giudice G, Crow MK (1993). Role of transforming growth factor beta (TGF*β*) in systemic autoimmunity. *Lupus*.

[38] Saverino D, Simone R, Bagnasco M, Pesce G (2010). The soluble CTLA-4 receptor and its role in autoimmune diseases: an update. *Autoimmunity Highlights*.

[39] de Carli M, D’Elios MM, Zancuoghi G, Romagnani S, del Prete G (1994). Human Th1 and Th2 cells: functional properties, regulation of development and role in autoimmunity. *Autoimmunity*.

[40] Saverino D, Merlo A, Bruno S, Pistoia V, Grossi CE, Ciccone E (2002). Dual effect of CD85/leukocyte Ig-like receptor-1/Ig-like transcript 2 and CD152 (CTLA-4) on cytokine production by antigen-stimulated human T cells. *Journal of Immunology*.

